# Association of globulin concentrations with prognosis in horses with lymphoma

**DOI:** 10.3389/fvets.2022.1086010

**Published:** 2023-01-09

**Authors:** Fiona M. Wensley, Emily H. Berryhill, K. Gary Magdesian

**Affiliations:** Department of Medicine and Epidemiology, University of California, Davis, Davis, CA, United States

**Keywords:** equine, globulin, hypoglobulinemia, lymphoma, lymphosarcoma, T cell, B cell

## Abstract

**Introduction:**

Lymphoma is the most common hemopoietic neoplasia in horses. Common clinicopathologic abnormalities in equine lymphoma include hyperglobulinemia, hypoalbuminemia, hyperfibrinogenemia, anemia, thrombocytopenia and lymphocytosis. Hypoglobulinemia has been reported in other species with lymphoma, however it has not been well-described in horses. The objectives of this study were to examine the prevalence of hypoglobulinemia in equine lymphoma, and to identify prognosis and clinicopathological abnormalities associated with serum globulin concentrations.

**Methods:**

Ninety-six horses with lymphoma were investigated in this retrospective study. Patients were allocated into groups based on serum globulin concentration. Survival analysis was performed to determine risk factors associated with globulin concentration and outcome.

**Results:**

Nineteen horses were hypoglobulinemic (≤2.1 g/dL), 63/98 were normoglobulinemic (2.2–4.3 g/dL), and 16/98 were hyperglobulinemic (≥4.4 g/dL). Hyperglobulinemia was associated with a higher anion gap (*P* = 0.0005), lower bicarbonate (*P* = 0.006), sodium (*P* = 0.03) and chloride concentrations (*P* = 0.002), and higher total protein than hypoglobulinemic horses (*P* < 0.0001). For location, 37% of horses with mucocutaneous lymphoma were hypoglobulinemic, compared to none in the hyperglobulinemic group (*P* = 0.02). Survival times were significantly different between low, normal and high globulin groups (*P* = 0.0002, median [range] survival times: 333 [1–3792], 43 [1–4,001] and 4 [1–129] days, respectively). Significant risk factors for shortened time to death were hyperglobulinemia (HR 2.4, *P* = 0.02), T cell lymphoma (HR 3.5, *P* < 0.0001), and multicentric (HR 3.1, *P* = 0.0008) and mediastinal (HR 6.4, *P* = 0.006) forms of lymphoma. Lack of chemotherapy was associated with shortened survival time (HR 4.5, P < 0.0001). B cell lymphomas (*P* < 0.0001) and mucocutaneous lymphoma location (*P* < 0.0001) were associated with longer survival times.

**Discussion:**

Serum globulin concentrations are associated with location of lymphoma, clinicopathologic abnormalities, and survival times in equine lymphoma.

## 1. Introduction

Lymphoma is the most common hematopoietic neoplasia encountered in horses and arises in lymph nodes and lymphoid tissue outside of the bone marrow, although metastasis may lead to bone marrow involvement (1–4). Equine lymphomas are a heterogenous group of lymphocytic malignancies and their classification relies on morphologic, immunophenotypic, genetic and clinical features (1, 5, 6). Hyperglobulinemia has been well-described in equine lymphoma (5, 7–11). The most common clinical chemistry abnormalities identified in one study were hyperfibrinogenemia (70%), hypoalbuminemia (51%) and hyperglobulinemia (35%) (5). Serum globulin concentrations comprise the alpha, beta and gamma globulin fractions. Gamma globulins account for the largest proportion of total globulin concentration, also known as immunoglobulins and secreted by B cells as part of the adaptive immune response (12). Low serum immunoglobulin concentrations, specifically IgM, have been evaluated as a diagnostic tool for equine lymphoma (13). Although a significant correlation between low IgM concentrations and lymphoma was not demonstrated, this study identified a subset of lymphoma patients with decreased immunoglobulin concentrations, either due to altered synthesis or increased catabolism (13). Hypoglobulinemia in horses may develop as a result of primary causes linked to genetic immune deficiencies, or secondary causes related to malignancies or their treatment, infections, medications and protein-losing states (12).

To the authors' knowledge, there are no studies investigating hypoglobulinemia or hyperglobulinemia and associations with type of lymphoma or prognosis in cases of lymphoma in horses. The objectives of this study were to examine the prevalence of hypoglobulinemia in horses diagnosed with lymphoma, and identify prognosis and describe hematological and biochemical parameters associated with serum globulin concentrations in lymphoma cases. The study hypothesis was that equine lymphoma patients with hypoglobulinemia or hyperglobulinemia have a distinct clinical profile and outcome.

## 2. Materials and methods

### 2.1. Case selection

A retrospective study was conducted using the medical records of adult horses admitted to the William R. Pritchard Veterinary Medical Teaching Hospital, University of California, Davis, USA, between 1 January 1998 and 2 May 2021. The Veterinary Medical and Administrative Computing System at UC Davis was searched using the keywords “lymphoma” and “lymphosarcoma.” Patients were included in the study if a diagnosis of lymphoma was made histopathologically from biopsies or at post mortem examination, and the patients had a globulin value and complete blood count submitted at the time of or after the diagnosis of lymphoma. Horses were excluded if the diagnosis was presumptive and not confirmed by histology. Definitive diagnosis of lymphoma was achieved by microscopic evaluation of biopsies or post mortem tissue samples. Lymphoma lineage was determined with either immunocytochemical or immunohistochemical immunophenotyping. Immunophenotypic assignment was based on an estimate of the percentage of positively staining cells for T and B cell markers (CD79a, CD3, and CD20) and the morphology and distribution of stained cells. For clonality conformation, or when immunophenotyping was not available, molecular clonality PCR for Antigen Receptor Rearrangements (PARR) assay was used to assign T or B cell lineage. Clonality assessment of genomic DNA was performed using capillary electrophoresis (14). Lymphocyte antigen receptor genes included IgH2, IgH3, KDEi (B cell), and TCRγ (T cell) to visualize DNA amplification rearrangements and generate a distinct clonal peak. A lineage was assigned (T cell or B cell) if there was a clear, reproducible clonal spike in the assigned lineage, with absence of a clonal spike in the PCR assay for the other lineage. A veterinary adaption of the World Health Organization (WHO) lymphoma classification system was applied for lymphoma categorization (6, 15). For horses that met the case inclusion criteria, the following data were evaluated from the medical record: signalment, date of admission, lymphoma type and anatomic location, clinical signs, complete blood count and serum biochemistry analysis panel, recent surgery or medical treatment with corticosteroids, chemotherapeutics or radiation therapy, discharge status (alive or dead), and if euthanized, the reason for euthanasia (owner's financial limitations vs. unfavorable prognosis or quality of life). For discussion and statistical analysis in this study, systemic chemotherapy incorporates systemic chemotherapeutic agents and corticosteroids used for immunosuppressive lymphoma treatment. Lymphoma was categorized according to 7 anatomical locations: gastrointestinal, mucocutaneous, hepatosplenic, mediastinal, central nervous system, ocular and multicentric (involving at least 2 organs, excluding the regional lymph node, as previously described) (6). Mucocutaneous lymphoma included skin, nasal mucosa and/or nasopharyngeal lymphoma. Location classification was based on either necropsy, when available, or staging, however full staging diagnostics were not required for inclusion. Diagnosis date was defined as date of confirmatory biopsy submission. The records were evaluated for follow-up information and owners were contacted *via* phone or email if no follow-up information was available, and the patient's discharge status was listed as alive. Survival times were defined as the time between diagnosis and the date of death or date of first owner contact (July 31, 2021) for those still alive at the time of analysis.

### 2.2. Grouping by globulin concentrations

A commercial chemistry analyzer determined globulin concentrations by calculating and deducting albumin from total protein concentration measurements using colorimetric assays (Cobas C Series Analyzer, Roche Diagnostics, Indianapolis, 46,256) (16, 17). Serum globulin measurements were preferentially used. When protein measurements were run on heparinized plasma (sodium heparin vacutainer) rather than serum, a corrected globulin value was calculated, subtracting the fibrinogen value in g/dL from the plasma globulin concentration (18). Plasma total protein was corrected for with a similar calculation. Cut-off points for normal globulin and total protein reference ranges were adapted from a study by Lumsden et al. (19). A normal globulin reference range of 2.2–4.3 g/dL (19) was used to categorize the patients into low (≤2.1 g/dL), normal (2.2–4.3 g/dL) and high (≥4.4 g/dL) globulin groups. A normal reference range of 2.7–3.4 g/dL was used for albumin concentration (19). A normal total protein reference range of 5.3–7.4 g/dL was used (19), to divide patients into low (<5.3 g/dL), normal (5.3–7.4 g/dL) and high (>7.4 g/dL) groups.

### 2.3. Statistical analyses

Descriptive statistics were used to report clinicopathologic findings. Numerical values are reported as medians and ranges for data that were not normally distributed, and means with standard deviation for normally distributed data. Data were tested for normality using a Kolmogorov-Smirnov test. A one-way ANOVA with Tukey *post-hoc* test for multiple comparisons was used to evaluate for differences in age and normally distributed clinicopathologic data among the low, normal and high globulin groups. Clinicopathologic data that were not normally distributed were compared using a Kruskal-Wallis test with Dunn post-test for multiple comparisons. Type and site of lymphoma were compared among the three groups using Chi-squared tests. Lymphoma cases with unknown immunophenotypes were excluded from association analysis.

Survival time from diagnosis to death was compared among the three globulin groups using a Kaplan-Meier survival analysis. Patients that were alive or lost to follow up at the end of the study period were right-censored at the time-point of last follow up. This was followed by a multivariable Cox proportional hazards regression analysis of two models to determine risk factors associated with globulin concentration and lymphoma outcome. The first model incorporated time from lymphoma diagnosis to globulin measurement and the second model included if patients were administered any systemic treatment prior to globulin measurement, these variables were applied to separate models due to the high likelihood of correlation. Both Cox regression models included survival analysis of the low, normal and high globulin and total protein groups, systemic treatment following lymphoma diagnosis, lymphoma type and anatomic location. The reference levels for the Cox regression comparison were chosen based on significance (*P* < 0.05) and median survival time from the Kaplan-Meier analyses, allowing for the use of a base comparison with the lowest likelihood of association with the event (death). To assess for any confounding effect on prognosis of total protein as a binary variable to globulin, patients were allocated into groups according to their total protein values, and survival analysis using Kaplan-Meier and Cox proportional hazards regression methods, were repeated. Significance was set at *P* < 0.05. The statistical software used were GraphPad Instat Software 3.10 and GraphPad Prism 9.4.1 (San Diego, CA).

## 3. Results

Ninety-six horses were identified that met the inclusion criteria and were included in the statistical analyses. There were 34 females and 62 males (*n* = 55 geldings, *n* = 7 stallions) ranging in age from 3 to 30 years (mean: 16.2 ± 6.8). Demographics for cases are shown in Table 1. There were no statistical differences in sex, breed or age among the low, normal and high globulin groups.

**Table 1:** Demographics of low, normal and high globulin groups.

	**Low globulin values** **(*n* = 19)**	**Normal globulin values** **(*n* = 61)**	**High globulin values** **(*n* = 16)**	**Total**
**Age (years)**				
Mean and SD (range)	14.8 ± 6.9 (4–29)	16.3 ± 7.2 (3–30)	17.2 ± 5.3 (8–25)	16.2 ± 6.8 (3–30)
**Breed:** ***n*** **(%)**				
Arabian	1 (5%)	7 (11%)	2 (14%)	11
Morgan	3 (16%)	1 (2%)	1 (7%)	5
Paint	3 (16%)	4 (6%)	1 (7%)	8
Quarter horse	8 (42%)	20 (32%)	4 (29%)	32
Thoroughbred	2 (11%)	15 (25%)	3 (21%)	20
Warmblood	2 (11%)	3 (5%)	3 (21%)	8
Other (Appaloosa, Crossbred, Draft, Mustang, Tennessee Walking Horse, Trakehner)	0 (0%)	12 (20%)	0 (0%)	12
**Sex:** ***n*** **(%)**				
Female	9 (47%)	20 (32%)	5 (36%)	34 (35%)
Gelding	9 (47%)	36 (59%)	9 (64%)	55 (57%)
Stallion	1 (5%)	6 (10%)	0 (0%)	7 (8%)

Differentiation of globulin concentration, anatomic location and lymphoma type is shown in Table 2. Globulin values were available for 75 patients prior to or at the initial visit for diagnosis of lymphoma; the remaining 21 patients had recorded globulin values after the diagnosis (median of 69 days from initial diagnosis visit). Nineteen horses were classified as hypoglobulinemic (≤2.1 g/dL), 61/96 as normoglobulinemic (2.2–4.3 g/dL), and 16/96 as hyperglobulinemic (≥4.4 g/dL). The majority of the cases were anatomically categorized as multicentric (52%), followed by mucocutaneous (27%), gastrointestinal (5%) and ocular (5%). Other anatomic locations included hepatosplenic (4%), central nervous system (3%), and mediastinal (3%). The most common lymphoma type was B cell (55%), with 35 cases (36%) classified as the subtype T-cell rich, B cell lymphoma, and 7 as diffuse large B cell lymphoma sub-types. Thirty-seven cases (39%) were classified as T cell lymphoma, including the sub-types hepatosplenic T cell (6%), large, granular T cell (6%), and B cell rich, T cell (1%). Six cases were categorized as unclassified lymphoma due to not having had immunohistochemistry (IHC) performed (*n* = 4), a clonal population unidentified by PCR for receptor antigen rearrangement assay (clonality) (*n* = 1) or classification as non B-cell and non T-cell lymphoma (*n* = 1).

**Table 2:** Globulin concentrations, lymphoma type, anatomic location and survival among low, normal and high globulin groups. Values reported as median (range) or *n* (%).

	**Low globulin values (*n* = 19)**	**Normal globulin values (*n* = 61)**	**High globulin values (*n* = 16)**	**Total (*n* = 96)**	**Median survival in days (range)**
**Globulin values (g/dL)**					
Median and range	1.9 (1.2–2.1)	3.3 (2.2–4.3)	4.7 (4.4–8.9)	3.2 (1.2–8.9)	
**Lymphoma type:** ***n*** **(%)**					
B-Cell	10 (53%)	33 (54%)	10 (63%)	53 (55%)	290 d (1–4001)
T cell rich large B-cell (TCRLBCL)	10 (53%)	23 (38%)	2 (13%)	35 (36%)	
Diffuse large B-cell	0	3 (5%)	3 (19%)	6 (6%)	
T-Cell	8 (42%)	24 (39%)	5 (31%)	37 (39%)	6 d (1–1,674)
Large granular T-cell	2 (11%)	4 (7%)	0	6 (6%)	
B cell rich T-cell	0	1 (2%)	0	1 (1%)	
Hepatosplenic T-cell	1(5%)	3 (5%)	2 (13%)	6 (6%)	
Cutaneous T-cell (CTCL)	1 (5%)	2 (3%)	0	3 (3%)	
Enteropathy associated T-cell (EATCL)	0	3 (5%)	1 (6%)	4 (4%)	
Unclassified	1 (5%)	4 (7%)	1 (6%)	6 (6%)	-
**Anatomic location:** ***n*** **(%)**					
Multicentric	11 (58%)	28 (46%)	11 (69%)	50 (52%)	6.5 d (1–4001)
Gastrointestinal	1 (5%)	3 (5%)	1 (6%)	5 (5%)	9 d (1–33)
Mucocutaneous	7 (37%)	19 (31%)	0	26 (27%)	957 d (13–3,792)
Ocular	0	4 (7%)	1 (6%)	5 (5%)	348 d (28–2,293)
Central Nervous System	0	2 (3%)	1 (6%)	3 (3%)	15 d (1–21)
Mediastinal	0	3 (5%)	0	3 (3%)	2 d (2–151)
Hepatosplenic	0	2 (3%)	2 (13%)	4 (4%)	11.5 d (1–129)
**Systemic chemotherapeutics administered:** ***n*** **(%)**					
Corticosteroids (prednisolone, dexamethasone)	14 (74%)	37 (60%)	5 (31%)	56 (58%)	151 d (1–4001)
Antimetabolite (Azathioprine)	1 (5%)	0	1 (6%)	2 (2%)	14 d (1–27 d)
Alkylating agents (cyclophosphamide, lomustine)	2 (11%)	0	1 (6%)	3 (2%)	1120.5 d (51–2,190)
Anthracyclines (doxorubicin)	4 (21%)	3 (5%)	1 (6%)	8 (8%)	-
Alkaloids (vincristine)	4 (21%)	2 (3%)	1 (6%)	7 (7%)	1957 d (9–3792)
L-asparaginase	1 (5%)	1 (2%)	0	2 (2%)	96.5 d (9–184)
**Chemotherapeutics administered prior to globulin measurement:** ***n*** **(%)**	4 (21%)	12 (20%)	1 (6%)	17 (18%)	578 d (1–3,792)
**Long term survival:** ***n***					
Alive	3	2	0	5	
Euth/Died	15	52	16	83	
Unknown	1	7	0	8	
**Duration of survival (Days)**					
Median and range	333 (1–3,792)	43 (1–4001)	4 (1–129)	33 (1–4001)	

Fifty-six (58%) of the horses received treatment with systemic immunosuppressants such as corticosteroids, either as monotherapy (*n* = 39) or in combination with other immunosuppressive drugs (*n* =16). Forty-two patients received dexamethasone, twenty-nine were treated with prednisolone and two received azathioprine. Intralesional corticosteroid injection with triamcinolone acetonide or methylprednisolone acetate, or topical dexamethasone sodium phosphate application was administered in eight patients. Ten patients received systemic chemotherapy with doxorubicin (*n* = 8), vincristine (*n* = 6), lomustine (*n* = 1), l-asparaginase (*n* = 2), and cyclophosphamide (*n* = 2). Three patients received intralesional chemotherapy with cisplatin (*n* = 1), bleomycin (*n* = 1), or mitomycin (*n* = 1). Six horses had surgical resections, including removal of cutaneous masses (*n* = 1), ocular masses (*n* = 4) and one had partial surgical resection of a segmental transmural colonic mass diagnosed as lymphoma. Two horses underwent radiation therapy. Of the 21 patients (22%) in which a lymphoma diagnosis was made prior to serum globulin measurements, 17 of these had treatment with chemotherapeutics (*n* = 16 corticosteroids; *n* = 1 doxorubicin) before recorded globulin values.

No significant differences in age were found among the three groups. There were also no differences in type (B vs. T cell lymphoma), or in multicentric, ocular, hepatosplenic, and mediastinal locations among groups. There was a significant difference among groups for mucocutaneous forms of lymphoma, in which 37% (7/19) were classified as having low globulins, 31% (19/61) as normal, and 0% (0/16) as high globulins (*P* = 0.03). A significant difference in lymphocyte counts was not found among horses with low, normal and high globulin values. Further, statistical differences between the remaining CBC parameters, including fibrinogen, total white blood cell count, monocytes, eosinophils, neutrophils, unclassifiable cells and all red blood cell parameters, were not found among groups.

Biochemistry parameters were investigated and showed significant differences in anion gap, bicarbonate, chloride, sodium and total protein concentration among groups, these results are listed in Table 3. Anion gap was significantly higher in the normal vs. low, and high vs. low groups. Bicarbonate concentrations were significantly higher in the low vs. high groups. Chloride concentrations were significantly higher in the low vs. high and normal vs. high groups. Sodium concentrations were statistically different between normal and high globulin groups. Not surprisingly, total protein concentration was significantly higher in the high globulin group as compared to the low and normal globulin groups. Albumin was inversely related to globulin, but was not statistically different among the three groups. There were a total of 42 horses with hypoalbuminemia (1.1–2.6 g/dL); 6 hypoalbuminemic horses in the low globulin group (1.1–2.6 g/dL), 26 in the normal globulin group (1.2–2.6 g/dL), and 10 in the high globulin group (1.7–2.6 g/dL). Differences in other electrolytes were not found to be statistically different when comparing groups (magnesium, potassium, phosphorus, calcium), as well as other biochemistry parameters (creatinine, BUN, glucose, AST, creatinine kinase, ALP, GGT, SDH, total bilirubin, triglycerides). Biochemistry panels were run on 53 serum samples and 43 plasma samples. Venous blood gases (VBG) were inconsistently measured among horses; only 19/96 cases had a VBG performed not in association with a general anesthetic, therefore statistical differences were not analyzed between groups. Of the 19 VBG results, the median lactate was 1.50 mmol/L with a range of 0.5–16 mmol/L.

**Table 3:** Significant clinical pathology findings among low, normal and high globulin groups.

**Biochemistry parameters**	**Low globulin values (*n* = 19)**	**Normal globulin values (*n* = 61)**	**High globulin values (n = 16)**	
**Anion gap (mmol/L)**				
Median (range)	12 (5-30) ^a^	15 (4-28) ^a, b^	19.5 (7-30) ^b^	*^*a*^ P* = 0.011, *^*b*^ P* = 0.0005
**Bicarbonate (mmol/L)**				
Median (range)	28.5 (12-33)^a^	26 (18–35)	24.5 (17–31) ^a^	^a^ *P* = 0.007
**Chloride (mmol/L)**				
Mean, SD	99.1 ± 3.8 ^a^	97.4 ± 4 ^b^	93.6 ± 4.4 ^a, b^	^a^ *P* = 0.0003, ^b^ *P* = 0.003
**Sodium (mmol/L)**				
Mean, SD	135.5 ± 2.7	135.5 ± 3.6 ^a^	132.9 ± 3.9 ^a^	^a^ *P =* 0.02
**Total protein (g/dL)**				
Median (range)	4.8 (3.0–5.8) ^a, c^	5.9 (4.4–7.4) ^b, c^	7.5 (6.1–11.2) ^a, b^	^a, b, c^ *P* < 0.0001

Numerical values are reported as medians and ranges for data that were not normally distributed, and means with standard deviation for normally distributed data. Significant *p*-values of < 0.05 between groups are indicated by letters.

**Figure 1:**
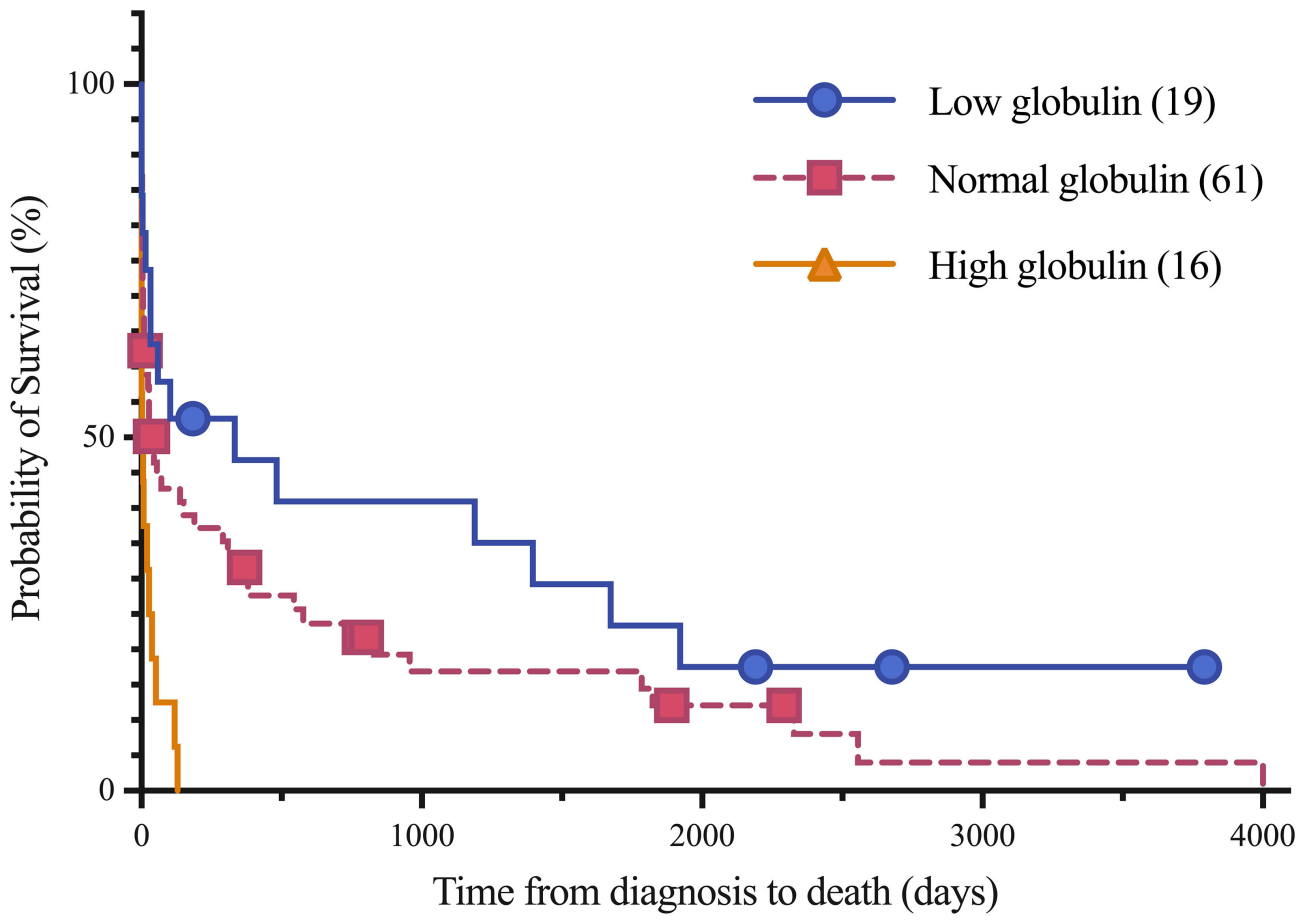
Kaplan-Meier analysis of survival time between low, normal and high globulin groups. Symbols plotted at censored points. Number of horses including censored patients (*n*). Comparison of survival curves *P*-value = 0.0002.

**Figure 2:**
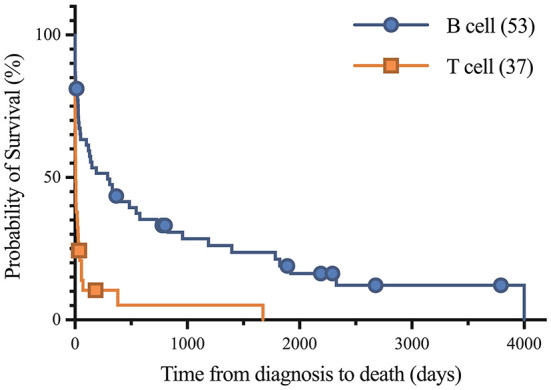
Kaplan-Meier analysis of survival time between B cell and T cell lymphoma. Symbols plotted at censored points. Median survival of B cell and T cell lymphoma were 290 and 6 days, respectively. Number of horses including censored patients (*n*). Comparison of survival curves *P*-value < 0.0001.

**Figure 3:**
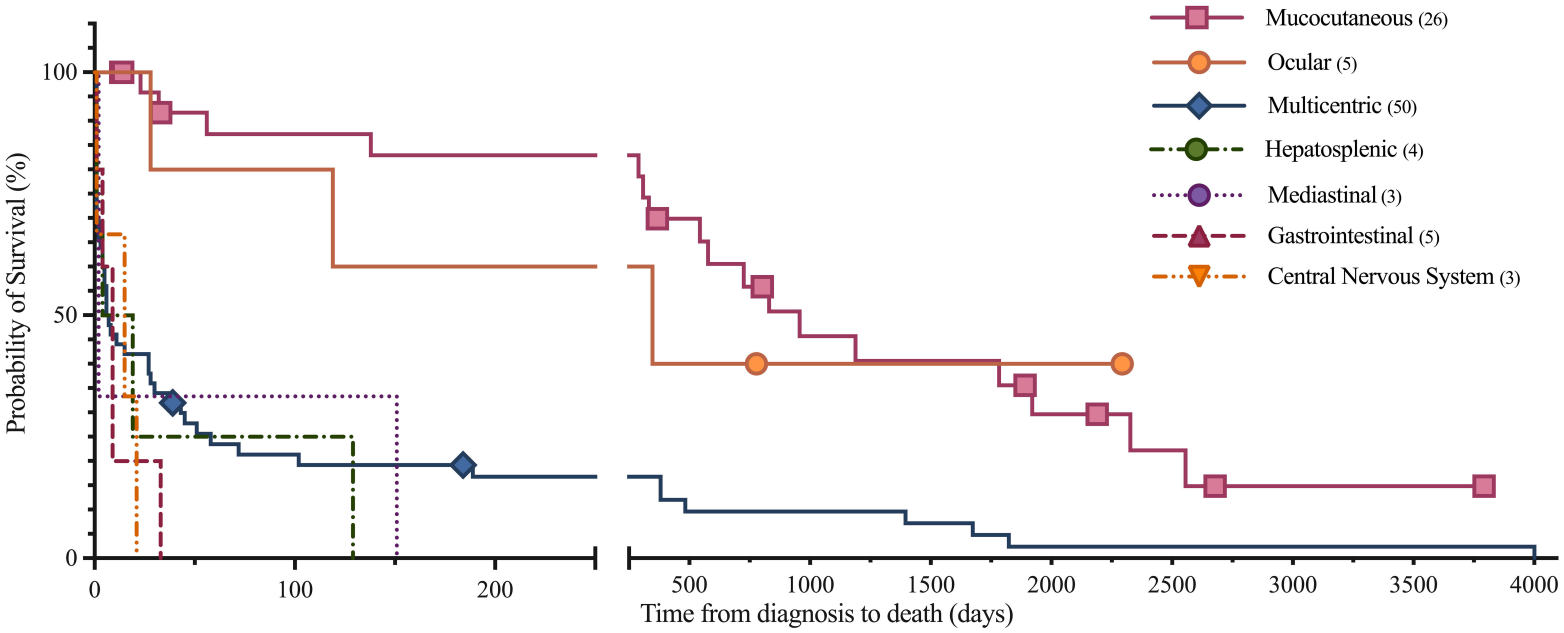
Kaplan-Meier analysis of survival time between anatomic locations of lymphoma. Symbols plotted at censored points. Number of horses including censored patients (*n*). Comparison of survival curves *P*-value < 0.0001.

**Figure 4:**
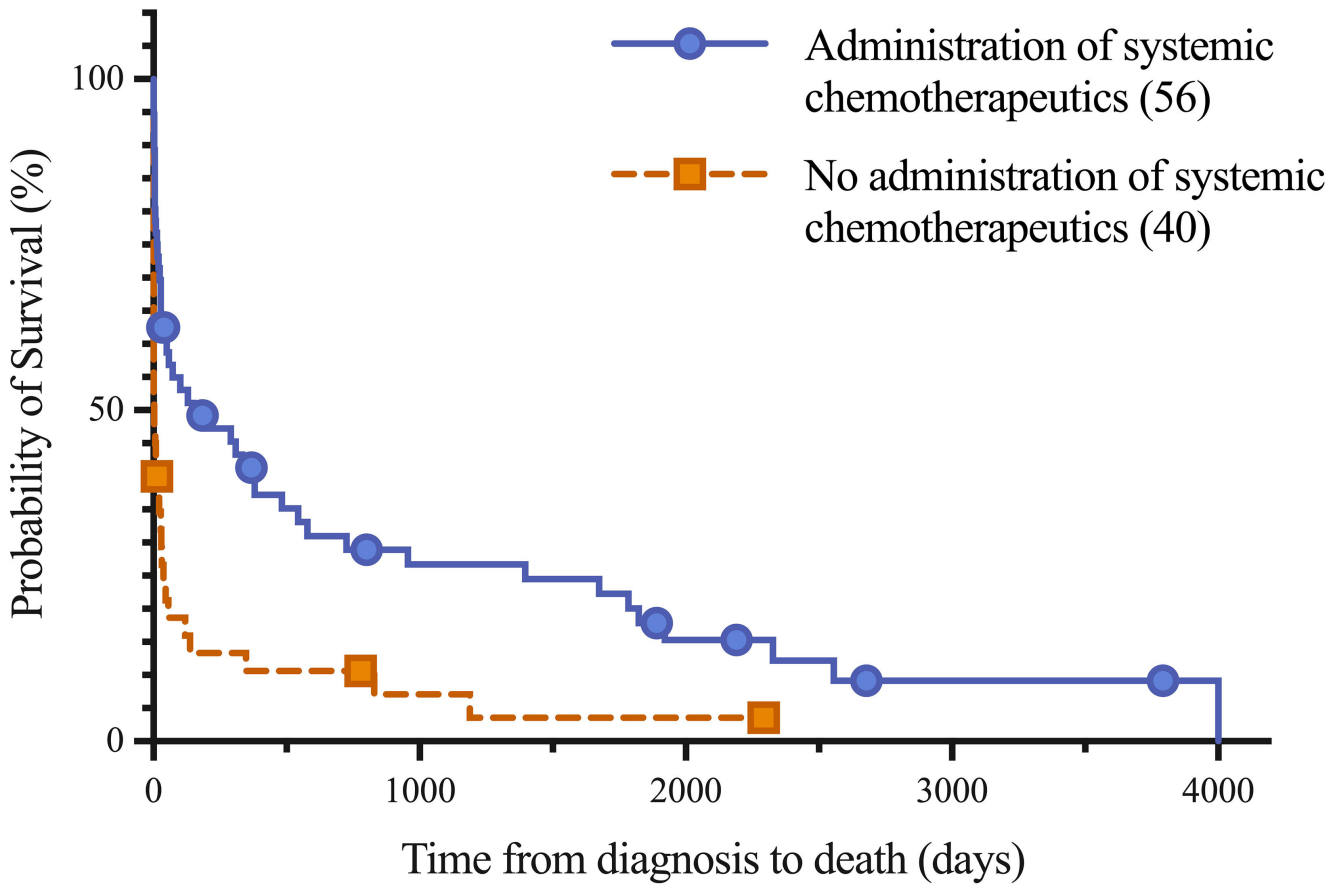
Kaplan-Meier analysis of survival time between horses treated with systemic chemotherapeutics, including corticosteroids, and untreated horses following lymphoma diagnosis. Median survival of treated and untreated lymphoma patients were 151 days and 4 days, respectively. Symbols plotted at censored points. Number of horses including censored patients (*n*). Comparison of survival curves P-value < 0.0001.

Short and long-term status following lymphoma diagnosis and hospital discharge were compared among low, normal and high globulin groups. Short-term was defined as the status (alive, euthanized or died) at the time of discharge from the last appointment at the referral hospital following lymphoma diagnosis. For short term survival, 35 horses were discharged alive and 61 did not survive to discharge from the hospital (euthanised, *n* = 60; died, *n* = 1). No significant differences were identified among groups for short term survival. Long-term status was defined as time from diagnosis to last known status at follow up with a set date in July 2021 for the alive horses, this ranged from 1 to 4,001 days with a median of 33 days. At this time, 5 horses were alive, 83 had not survived (euthanized, *n* = 82; died, *n* = 1), and 8 were lost to follow up with unknown outcomes. A postmortem examination was completed on 52 of the 83 deceased horses. Survival time, with the alive and lost to follow-up horses censored, was significantly different between all three globulin groups (*P* = 0.0002), shown in Figure 1. The high globulin group (median: 4 days; range 1–129 d) had a significantly shorter duration of survival from time of diagnosis as compared to the low globulin (median: 333 days; range 1–3,792 d; *P* = 0.0003) and normal globulin (median: 43 days; range 1–4,001 d; *P* = 0.0005) groups. The low and normal globulin groups were not statistically different in duration of survival. When only considering horses treated with chemotherapy, these results were similar; the high globulin group (median: 27 days; range: 2–129 d) had significantly shorter survival times compared to low (median: 483 days; range: 1–3,792 d; *P* = 0.006) and normal (median: 189 days; range: 1–4,001 d; *P* = 0.03) groups. The low and normal groups were not statistically different. A summary of Kaplan-Meier survival estimates is described in Table 4. Kaplan-Meier survival analyses of lymphoma type, location and treatment are depicted in Figures 2–4, respectively. Cox proportional hazard regression models (hazard ratios and confidence intervals reported in Tables 5, 6) revealed a significantly increased hazard ratio of 2 in the high globulin group in comparison to the patients with a normal globulin (*P* = 0.02). T cell lymphoma had an increased hazard ratio of 3 in comparison to B cell lymphoma (*P* < 0.0001). If no systemic chemotherapy was administered following the diagnosis of lymphoma, this was associated with a hazard ratio of 4 (*P* < 0.0001), increasing the likelihood of reaching an endpoint of death earlier than horses that did receive treatment with systemic chemotherapy. The two Cox regression models detected no significant effects of timing of globulin measurement in relation to lymphoma diagnosis nor of systemic chemotherapy having been administered prior to globulin measurement. Age at exam was not significant in the Cox regression models, therefore was removed to reduce the number of variables. The groups were readjusted for total protein survival analysis. There were 21 horses in the low total protein group (median: 4.7 g/dL; range: 3–5.2 g/dL), 67 horses with a normal total protein (median: 6 g/dL; range: 5.3–7.4 g/dL) and 8 horses in the high total protein group (median: 7.9 g/dL; range: 7.6–11.2 g/dL). Kaplan-Meier analysis demonstrated median survival times of 33, 45, and 14.5 days in the low, normal and high total protein groups, respectively. There was no statistical difference between all three groups for survival, or individually between low and normal, or low and high groups. There was a statistically significant difference of survival between the normal (median: 45 d, range: 1–4,001 d) and high (median: 14.5 d, range: 1–129 d) total protein groups (*P* = 0.03). No statistical significance in survival was found when the Cox regression models were repeated using the total protein groups in place of globulin.

**Table 4:** Summary of Kaplan-Meier estimates for survival of 96 horses with lymphoma.

**Time (Years)**	**Survival (%)**	**Number of deaths**	**Number censored**	**Number at risk**
0.2	40.6	55	4	37
0.5	34.9	59	5	32
1	29.2	64	6	26
2	21.8	71	6	19
5	12.3	78	8	10
10	6.4	83	12	3

**Table 5:** A Cox proportional hazards regression model showing the effect of eleven variables on the risk of death, including if systemic chemotherapeutics were administered prior to globulin measurement (model 1).

**Variable**	**Coefficient; β (95% confidence interval)**	**Hazard ratios (95% CI)**	***P*-value**
**Globulin:**			
Normal (reference)	-	-	-
Low	−0.63 (−1.32 to −0.002)	0.53 (0.27 to 1.0)	0.06
High	0.87 (0.14 to 1.60)	2.39 (1.16 to 4.80)	0.02
**Systemic chemotherapeutics administered prior to globulin measurement:**			
No history of treatment (reference)	-	-	-
History of treatment	0.24 (−0.49 to 1.0)	1.27 (0.62 to 2.50)	0.5
**Systemic chemotherapeutics administered following lymphoma diagnosis:**			
History of treatment (reference)	-	-	-
No history of treatment	1.62 (1.02 to 2.24)	5.09 (2.78 to 9.42)	< 0.0001
**Lymphoma type:**			
B cell (reference)	-	-	-
T cell	1.33 (0.75 to 1.90)	3.77 (2.13 to 6.71)	< 0.0001
**Anatomic location:**			
Mucocutaneous (reference)	-	-	-
Multicentric	1.12 (0.48 to 1.80)	3.06 (1.61 to 6.01)	0.0008
Gastrointestinal	1.32 (0.03 to 2.50)	3.75 (0.97 to 12.03)	0.03
Ocular	−1.21 (−2.70 to −0.04)	0.30 (0.07 to 0.96)	0.07
Central nervous system	1.30 (−0.26 to 2.60)	3.66 (0.77 to 12.8)	0.06
Mediastinal	1.85 (0.34 to 3.04)	6.35 (1.40 to 21.0)	0.006
Hepatosplenic	1.50 (0.13 to 2.68)	4.50 (1.13 to 14.52)	0.02

A hazard ratio below or above 1, and excluding 1 in the 95% confidence interval, and with a *p-*value of < 0.05 are clinically significant.

**Table 6:** A Cox proportional hazards regression model showing the effect of eleven variables on the risk of death, including 21 patients that had a globulin measurement following time of diagnosis (model 2).

**Variable**	**Coefficient; β (95% confidence interval)**	**Hazard ratios (95% CI)**	***P*-value**
**Globulin:**			
Normal (reference)	-	-	-
Low	−0.43 (−1.12 to 0.20)	0.65 (0.33 to 1.22)	0.2
High	0.96 (0.19 to 1.70)	2.61 (1.21 to 5.46)	0.01
Time from diagnosis to globulin measurement	−0.0008 (−0.002 to −0.00004)	1.0 (0.99 to 1.00)	0.07
**Systemic chemotherapeutics administered following lymphoma diagnosis:**			
History of treatment (reference)	-	-	-
No history of treatment	1.50 (0.90 to 2.11)	4.50 (2.50 to 8.20)	< 0.0001
**Lymphoma Type:**			
B cell (reference)	-	-	-
T cell	1.25 (0.65 to 1.87)	3.50 (1.91 to 6.50)	< 0.0001
**Anatomic location:**			
Mucocutaneous (reference)	-	-	-
Multicentric	1.29 (0.58 to 2.03)	3.62 (1.80 to 7.60)	0.0005
Gastrointestinal	1.44 (0.07 to 2.64)	4.22 (1.07 to 14.0)	0.02
Ocular	−1.21 (−2.71 to −0.03)	0.30 (0.07 to 1.0)	0.07
Central nervous system	1.0 (−0.96 to 2.40)	2.63 (0.38 to 10.90)	0.2
Mediastinal	2.0 (0.44 to 3.20)	7.13 (1.60 to 24.12)	0.004
Hepatosplenic	1.35 (−0.30 to 2.70)	3.84 (0.80 to 14.90)	0.07

Patients with a globulin measurement before time of diagnosis (day 1) were excluded from analysis.

A hazard ratio below or above 1, and excluding 1 in the 95% confidence interval, and with a *P*-value of < 0.05 are clinically significant.

## 4. Discussion

This retrospective study describes an uncommon finding of hypoglobulinema in horses diagnosed with lymphoma. The study demonstrated that hyperglobulinemia may be a negative prognostic indicator for long term survival in equine lymphoma. T cell lymphomas and multicentric and mediastinal lymphoma locations were associated with a decreased survival time as compared to B cell lymphomas and those located in other locations, respectively. An increased hazard ratio was also appreciated when no systemic chemotherapy was administered following diagnosis. Multicentric lymphoma was the most common presentation of the disease, similar to previous published reports (2, 4, 6). Diagnosis with B cell lymphoma and a mucocutaneous location of lymphoma were significantly associated with more favorable survival times.

Globulin concentrations were more strongly associated with prognosis than total protein in this analysis. Hyperglobulinemia may result in down regulation of albumin synthesis to help regulate colloid osmotic pressure and thus maintain the measured serum total protein or increase it to a lower amount than it would otherwise. A decreased albumin concentration may also be stimulated by a negative acute phase response secondary to inflammation or cancer cytokines, as well as possibly due to inadequate intake and absorption, increased loss or sequestration.

In this study, all of the B cell lymphomas diagnosed in horses in the low serum globulin group were classified as T-cell rich, large B cell lymphoma (TCRLBCL). Mucocutaneous lymphomas, which were most frequently TCRLBCL, similar to previous studies (6, 20), were highest in prevalence in the low globulin group (37%), fairly similar in the normal globulin group (31%), and absent in the high globulin group (0%). Similarly, the TCRLBC lymphomas were least common in the high globulin group consisting of 13% of tumors, in comparison to 53% in the low and 38% in the normal globulin groups. It is possible that TCRLBC lymphomas down regulate antibody production by normal lymphocytes, and this warrants further study, as does the effects of hypoglobulinemia on risk of infection. Consistent with this, one 13-year-old gelding in this study with mucocutaneous T-cell and histiocyte-rich B-cell lymphoma was diagnosed with common variable immunodeficiency (CVID) associated with multiple and recurrent infections (salmonella enteritis, pyelonephritis, pneumonia, intramuscular abscess, and fungal keratitis) and impaired antibody production with low serum IgM (<33 mg/dL; reference median 100 mg/dL + 50 mg/dL CI) and IgG (855 mg/dL; reference median 1,760 + 603 mg/dL CI) values.

Hypogammaglobulinemia at time of diagnosis in cases of chronic lymphocytic leukemia in human patients has been shown to predict for shorter time to first treatment intervals, independent of other risk factors (21). Additionally, humans with concurrent diffuse large B-cell lymphoma and low total gamma globulin levels had higher overall death rates and infection-related deaths as compared to those with higher total globulin concentrations (22). It is to be noted that total globulin concentrations were evaluated in this study rather than specific immunoglobulin concentrations. Use of intravenous immunoglobulin infusions in clinical trials for humans with hypoglobulinemia have demonstrated a reduction in the incidence of mild and moderate bacterial infections, but not a decrease in mortality (23). In contrast to what is reported in humans, and although the association between hypoglobulinemia and risk of infection is well-documented, in the current study, horses with hypoglobulinemia had longer survival times than those with hyperglobulinemia. One possibility for this finding is that 37% of the horses with hypoglobulinemia had lymphoma affecting mucocutaneous sites, as opposed to horses with hyperglobulinemia in which none of the animals had mucocutaneous lymphoma and 69% were affected by multicentric lymphoma. Mucocutaneous lymphoma may be more localized and is associated with a better prognosis, compared to multicentric disease. Further study investigating the differences between these disease processes is indicated.

Causes of hyperglobulinemia may reflect a paraneoplastic process or the abnormal antibody products of cancerous lymphocytes. In a small study evaluating characterization of B cell lymphomas in horses, all five patients had hyperglobulinemia (9). The hyperglobulinemia was due to IgG1 or IgG4/7 monoclonal gammopathy (9). Chemotherapy and chemoimmunotherapy tend to decrease immunoglobulin levels in humans over time and can magnify immunosuppression. No significant effect on globulin value or prognosis was identified in the 17 horses in which a chemotherapeutic agent was administered prior to globulin measurement in this study. However, treatment course timing and specific dosing regimens were not analyzed, therefore immunosuppressive treatment effect on globulin values in horses with lymphoma warrants further study. Specific treatment modalities were not statistically evaluated as treatment was not a requirement for case selection in this study and were quite variable among horses. Outcome of the 57 horses who received systemic chemotherapeutics following diagnosis of lymphoma was associated with longer survival times, when compared to the 41 horses who did not receive systemic chemotherapeutics. This is in agreeance with a previous clinical outcome study which demonstrated that chemotherapy can be used successfully for treatment of horses with lymphoma, with mild adverse effects (24).

Hypogammaglobulinemia has been identified in a multitude of lymphoid malignancies in human patients (21, 22, 25, 26). It has been most profound in patients with chronic lymphocytic leukemia, with up to 85% of patients being hypoglobulinemic, and in diffuse large B cell lymphoma (DLBCL), with a prevalence of 22% in newly diagnosed DLBCL patients (25, 26). This is similar to the high prevalence of large B cell lymphomas in the horses of the low globulin group in this study. Although the precise mechanism of immune deficiency is not clear, it is likely mediated by the presence of tumor cells within lymphoid organs affecting the interaction between B cells and T cells in the normal immune response (21). A lymphoma involving B cells may be unable to synthesize immunoglobulins or develop poor function as antigen presenting cells. A T-cell lymphoma may induce a state of T-cell anergy with poor responses in mixed lymphocyte reactions, Th2 polarization, and aberrant expression of intracellular adhesion molecules, causing a lack of or decrease in immunoglobulin production (21, 23).

There were differences in a few clinicopathologic variables among the groups in this study. Horses in the high and normal globulin groups had a significantly higher anion gap as compared to those in the low globulin group. Bicarbonate concentrations were significantly higher in the low vs. high groups and chloride concentrations were significantly higher in the low vs. high groups and normal vs. high groups. One possibility for the differences in anion gap is an artifactual effect of different protein concentrations on acid-base balance. A lower total protein concentration would be expected to lower anion gap, which may partially explain the decreased anion gap in the low globulin group (27). Alternatively, the increased anion gap and decrease in chloride and bicarbonate in the high and normal globulin groups may be a compensatory or expected decrease in response to increased unmeasured anions, especially lactate. The sodium concentrations followed a similar pattern to chloride levels, with a significant decrease in the high group in comparison to the normal group. The normal mean sodium-chloride difference maintained between groups is consistent with the presence of unmeasured anions in the high globulin group. The median anion gap for the high globulin group was 19.5 mmol/L, above the laboratory's normal reference range of 9–17 mmol/L. Unfortunately, plasma lactate was not consistently measured in these horses. Hyperlactatemia is a common cause of increased anion gap in horses and has been documented in human and canine lymphomas (28, 29). Malignancy-induced lactic acidosis is suspected to be associated with the high metabolic rate of rapidly growing tumors and cancer metabolic reprogramming, resulting in hypoxia and increased glycolysis and thus lactate production by cancer cells, potentially exceeding the normal clearance rate of muscle and liver lactic acid (29). Plasma lactate concentration was only measured in 19/98 cases in this study, not in association with a general anesthetic, therefore blood lactate values were not able to be statistically compared between groups. The median lactate was 1.45 mmol/L in these 19 horses, with a range of 0.5–16 mmol/L, indicating half of the horses with lymphoma that had lactate measured had concentrations above 1.5 mmol/L. This warrants further study of lactate dynamics in horses with lymphoma.

Limitations of this study included the relatively small sample size and the retrospective study design resulting in a reliance on medical records for complete case details. The effect on biochemistry parameter variations between serum and plasma samples was considered. However, studies have shown no significant differences in any analytes measured between serum and plasma except for potassium (6–9% difference) (30). It has been shown that correlation between corrected plasma total protein and serum total protein is high (*r* = 0.985), with a mean difference of 0.078 g/dL between the two after subtraction of fibrinogen in plasma samples (18). In the horses of this study, 78% were newly diagnosed with lymphoma with no recorded prior treatment at time of globulin measurement, and the remaining 22% of globulin values were taken at variable time points following lymphoma diagnosis. Chronicity or disease progression may have affected globulin response. The Cox regression models helped to reduce the possibility of bias of an effect of timing of and treatment prior to globulin measurement. There was no significant effect of treatment on globulin levels in the 17 horses known to have received chemotherapeutics prior to globulin measurement. Duration and degree of clinical signs prior to diagnosis and globulin measurement, as well as any treatment course or dose, were unreliably documented in the medical records, and therefore not able to be included in statistical analysis to determine an effect on globulin values. In addition, only 62% of the deceased horses underwent an autopsy, allowing for complete documentation of anatomic location and sampling of all systems and nodes to accurately diagnose the extent of the lymphoma. Although the majority of reasons recorded for euthanasia were demonstrated to be due to severity of clinical signs, deterioration with or without treatment, or predicted poor or grave prognosis, we are unable to completely exclude shortened survival affected by owner's perception of or preference for treating neoplasia, potentially resulting in bias. Euthanasia early after diagnosis as per owner decision may have resulted in shortened survival times in some of the horses.

In conclusion, horses with lymphoma that are hyperglobulinemic have higher serum anion gaps, lower bicarbonate, sodium and chloride concentrations, and higher total protein concentrations than horses with low globulins. A globulin concentration of >4.3 g/dL in equine lymphoma cases was associated with a statistically significant shorter duration of survival from time of diagnosis, median of 4 days, in comparison to low globulin (<2.2 g/dL) and normal globulin values (2.2–4.3 g/dL), with median survival times of 333 days and 43 days, respectively.

## Data availability statement

The original contributions presented in the study are included in the article/supplementary material, further inquiries can be directed to the corresponding author.

## Ethics statement

Ethical review and approval, as well as specific written informed consent, was not required for the animal study due to the retrospective nature of the study. Clients presenting animals to the hospital provide written consent for data collected from their animals to be used for future research and teaching material.

## Author contributions

FW and KM designed the study, interpreted and analyzed results, and wrote and revised the manuscript. FW conducted medical record search and data acquisition. KM statistically analyzed results. EB participated in manuscript writing and revision. All authors contributed to the article and approved the submitted version.
